# Multi-omic analyses of triptan-treated migraine attacks gives insight into molecular mechanisms

**DOI:** 10.1038/s41598-023-38904-1

**Published:** 2023-07-31

**Authors:** Lisette J. A. Kogelman, Katrine Falkenberg, Filip Ottosson, Madeleine Ernst, Francesco Russo, Valdemar Stentoft-Hansen, Samuel Demharter, Peer Tfelt-Hansen, Arieh S. Cohen, Jes Olesen, Thomas Folkmann Hansen

**Affiliations:** 1 Department of Neurology, Danish Headache Center, Copenhagen University Hospital, Glostrup, Denmark; 2 Department of Congenital Disorders, Section for Clinical Mass Spectrometry, Danish Center for Neonatal Screening, Statens Serum Institut, Copenhagen, Denmark; 3 Abzu ApS, Copenhagen, Denmark; 4 Novo Nordisk Foundation Center for Protein Research, University of Copenhagen, Copenhagen, Denmark

**Keywords:** Migraine, RNA sequencing, Time series, Systems analysis, Data integration, Drug regulation

## Abstract

Migraine is a common, polygenic disorder that is characterized by moderate to severe headache attacks. Migraine attacks are commonly treated with triptans, i.e. serotonin receptor agonists. However, triptans are effective in ~ 60% of the population, and the mechanisms of triptans are debated. Here, we aim to expose the mechanisms of triptan using metabolomics and transcriptomics in spontaneous migraine attacks. We collected temporal multi-omics profiles on 24 migraine patients, using samples collected at a migraine attack, 2 h after treatment with a triptan, when headache-free, and after a cold-pressor test. Differential metabolomic analysis was performed to find metabolites associated with treatment. Their effect was further investigated using correlation analysis and a machine learning approach. We found three differential metabolites: cortisol, sumatriptan and glutamine. The change in sumatriptan levels correlated with a change in *GNAI1* and *VIPR2* gene expression, both known to regulate cAMP levels. Furthermore, we found fatty acid oxidation to be affected, a mechanism known to be involved in migraine but not previously found in relation to triptans. In conclusion, using an integrative approach we find evidence for a role of glutamine, cAMP regulation, and fatty acid oxidation in the molecular mechanisms of migraine and/or the effect of triptans.

## Introduction

Migraine is a common polygenic disorder with a world-wide prevalence of 14.1%^1^ and a heritability estimates between 34 and 57%^2^. A migraine attack is characterized by a moderate to severe headache attack lasting for 4–72 h. The headache is unilateral, pulsating and/or aggravated by physical activity, and is accompanied by nausea, vomiting, and/or photophobia and phonophobia^3^. An effective acute treatment of migraine attacks are the triptans, a family of tryptamine-based drugs. Triptans are effective in approximately 60 per cent, based on headache response at 2 h, but only approximately 30% of the migraine patients is headache-free 2 h after taking a triptan^4^. Understanding the biological mechanisms of triptans may explain the lack of response in some migraine patients.

Triptans are hydrophilic, but cross the blood–brain barrier to some extend during a migraine attack^5^. Triptans are 5-HT_1B/1D_ receptor agonists, but their mode and site of action is debated^6^. The 5-HT_1B/1D_ receptors are G-protein coupled receptors, that are very similar but not identical. They inhibit adenylate cyclase which decreases cyclic adenosine monophosphate (cAMP) synthesis. They also inhibit neurotransmitter release by coupling to K^+^ and Ca^2+^ channels and stimulate nuclear extracellular signal-regulated kinases (ERK) translocation. They are expressed in serotonergic neurons and in the trigeminal ganglia where they co-localize with CGRP, substance P and NOS, all shown to have a role in migraine mechanisms, suggesting that triptans act in the central nervous system. However, the 5-HT_1B_ receptor mediates vasoconstriction, suggesting a peripheral effect.

The metabolome consists of several thousands of metabolites, which are the intermediate- and final products of metabolism. They are the result of interactions between gene/protein expression and environment, and it is the closest ‘omic level to the expressed phenotype. In combination with other ‘omics it has great potential to reveal disease and drug mechanisms by explaining how genes translate to function. To date, the effect of triptans on a spontaneous migraine attack using ‘omics data has never been investigated.

We collected blood samples during spontaneous migraine attacks, before and after acute treatment with a triptan. We analyzed (untargeted) metabolomics to find changes initiated by the migraine attack and/or the treatment and integrated with transcriptomics to map molecular mechanisms. We identified three metabolites differentially expressed after treatment: cortisol, sumatriptan, and glutamine. Integration with transcriptomics revealed key pathways involved in migraine and its treatment with a triptan.

## Methods

### Sample collection

Samples were collected of migraine patients suffering a spontaneous migraine attack, as published previously^7^. In short, we recruited 100 migraine patients (17 males, 83 females), which were diagnosed based on International Headache Society criteria, aged 18–70 years, weighing between 45 and 95 kg and of Danish ethnicity. None of them were pregnant, breastfeeding or having any recent change in daily medication. A full medical history was taken at the hospital, including an electrocardiography (ECG), physical examination, vital signs and a validated semi-structured headache questionnaire. Recruitment was done via the website “forsøgsperson.dk”, via the Danish Headache Centre, via Facebook and by advertising at hospitals.

### Study design

**Figure 1:**
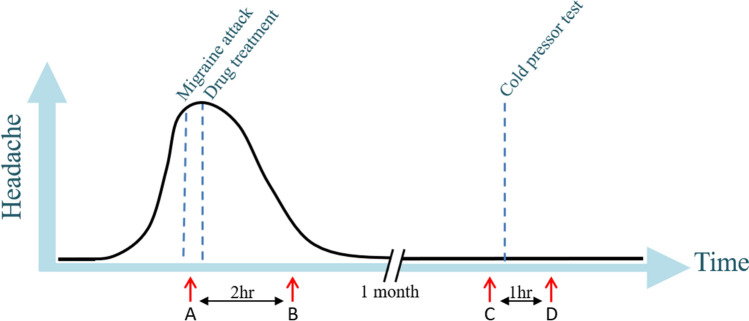
Study design with on the x-axis the time and on the y-axis the degree of headache. Blood sampling (marked by red arrows) was performed at four time points: (**A**) during migraine attack, (**B**) 2 h after treatment, (**C**) at a headache-free day and (**D**) after a cold-pressor test. Figure previously published by Kogelman et al.^7^.

The 100 migraine patients were instructed to contact the responsible doctor or medical student by phone at the onset of a migraine attack. This resulted in 24 migraineurs of which 17 had a migraine without aura (MO) attack and seven had a migraine with aura (MA) attack, all patients were females. The study is registered and described on clinicaltrials.gov (NCT02468622). Patients were instructed to come to the hospital by taxi, or the doctor/medical student went to the patient’s home during a spontaneous migraine attack. Upon arrival, a blood sample was taken from the cubital vein (time point A) and the patient was treated with subcutaneous sumatriptan; one patient chose to take rizatriptan (10 mg) tablet and one eletriptan (40 mg) tablet. Every half an hour, up to 2 h after treatment, attack-specific phenotype data was collected, including headache intensity (on the visual analogue scale [VAS], i.e., 0–10), headache characteristics and any associated symptoms. Two hours after treatment another blood sample was taken (time point B). Approximately a month after the migraine attack, ensuring similar time in the menstrual cycle, a blood sample was taken at the same time of the day, and at the same physical place, as the migraine attack (time point C). The patient had to be headache-free for at least 24 h and migraine-free for 5 days. Subsequently, to investigate the general pain/stress response, a cold pressor test was performed, by letting the subject keeping her hand for as long as tolerated, with a maximum of 10 min, in ice water. After the 60 min, another blood sample was taken (time point D). The study design is visualized in Fig. 1.

### Steroid profiling

The steroids 17-hydroxyprogesterone, testosterone, androstenedione and cortisol were measured as previously described^7^. To optimize normal distribution, cortisol was square root transformed and androstenedione, progesterone and testosterone were log transformed.

### Untargeted metabolomics

Metabolomic profiling was performed as previously described^8^, using samples of all four time-points simultaneously. Summarized, plasma samples were submitted to untargeted liquid chromatography-tandem mass spectrometry (LC–MS/MS) metabolomics measurements at Statens Serum Institute, Denmark. Data were preprocessed using MZmine v2.40.1 and chemical structural annotation was performed through the feature-based mass spectral molecular networking workflow within the Global Natural Products Social Molecular Networking Platform (GNPS)^9^^,^^10^. To further enhance chemical structural information, MS2LDA substructure information (https://ccms-ucsd.github.io/GNPSDocumentation/ms2lda/)^11^ and information from in silico structure annotation from Network Annotation Propagation^12^ were incorporated within the GNPS mass spectral molecular network using the MolNetEnhancer workflow (https://ccms-ucsd.github.io/GNPSDocumentation/molnetenhancer/)^13^. In addition, MS/MS fragmentation spectra were searched using the in silico tools SIRIUS + CSI:FingerID^14^ and CANOPUS^15^. Missing data were imputed as zeroes. Relative intensities were scaled by dividing each mass spectral feature by its batch root mean square. After batch normalization, no significant batch effect was observed using a permutational multivariate analysis of variance (P = 0.073, Adonis R2 = 0.0079). Features were filtered based on 20-fold difference between blank and experimental samples (based on their maximum intensity) and at metabolite-level, features were filtered based on the modified 80% rule (i.e., mass spectral features present in at least 80% of the samples per experimental group). This resulted in a dataset of 622 mass spectral features with associated MS/MS fragmentation spectrum, which for better readability we from now on refer to as metabolites. Only metabolites with at least a metabolite identification level 2 according to the Metabolomics Standard Initiative’s reporting standards^16^ were presented, meaning they required an accurate parent mass (m/z) with < 3 ppm error, high fragmentation pattern similarity (cosine > 0.8) to data in the public domain or in silico structure evidence from at least two independent tools (GNPS network, SIRIUS + CSI:FingerID, MS2LDA). Spectral mirror plots for differential and predictive metabolites with matches to GNPS spectral libraries are found in Supplementary Fig. 1.

### Transcriptomics

RNA-Sequencing was performed as previously described, using samples of all four time-points simultaneously. In short, blood samples were stored in PAXgene Blood RNA Tubes and RNA was extracted using the PAXgene Blood RNA Kit (Qiagen, Venlo, The Netherlands) by deCODE Genetics, Reykjavik, Iceland. RNA quality was checked using the Agilent 2100 Bioanalyzer and LabChip. RNA-Sequencing was performed on the Illumina Novaseq by deCODE Genetics, resulting in paired-end reads of 125 basepairs long. Files were processed and quantified with kallisto v0.42.5^17^ using the human reference transcriptome (Gencode Release 28). Using the R package tximport, transcript abundances were merged into gene abundances. Outliers were detected using the Mahalanobis' distance (MD) using the first eight principal components; a MD larger than the chi-square value for df = 8 at an alpha value of 0.01 were removed. Data was normalized for library size and gene-length bias using the DESeq2 package^18^ using the gene-length/sequencing-depth matrix estimated by kallisto. Genes that were not expressed in at least 90% of the samples were removed and only protein-coding genes were retained for analysis (n = 15,940).

### Statistical analyses

Differential metabolic analysis was performed using a Wilcoxon Rank Sum test. Metabolite levels were compared during migraine attack versus after migraine treatment, and before versus after the cold pressor test. *P*-values were corrected for multiple-testing using Benjamini & Hochberg (*P*_adj_). Metabolites were called as differential metabolites when *P*_adj_ < 0.05. Correlation analysis of identified differential metabolites with other metabolites and gene expression was calculated using Spearman’s correlation coefficients (presented as rho [r_s_]). Statistical analyses were performed in R (v4.1.2).

### Identification of predictive metabolites and genes with symbolic regression

In case no significant associations were found with relevant metabolites using a simple correlation analysis, we used a machine learning method based on symbolic regression called QLattice (v3.0.1) to identify metabolites and/or genes that are predictive of the detected differential metabolites^19^. Instead of calculating the association of only a single gene or metabolite at a time with the differential metabolite (i.e., correlation analysis), this approach reveals the combination of genes/metabolites that best "predict" the outcome (i.e., expression of the metabolite). The QLattice incorporates both linear and non-linear combinations. To limit overestimation of the prediction, we limited the number of features included in the model by using a derivative of the Bayesian Information Criterion. This approach penalizes complexity of the models and prevents overfitting, which is especially important when the data contain many more features than samples. 

### Ethics approval and consent to participate

The study was approved by the thics committee of Copenhagen (H-15006298), by the Danish Data Protection Agency (I-suite 03786). The study is registered on clinicaltrials.gov (NCT02468622) and was conducted according to the Helsinki II declaration of 1964, as revised in 2008. All participants gave written informed consent after receiving oral and written information.

## Results

A spontaneous migraine attack in 24 migraine patients was treated with triptan, of which 22 received subcutaneous sumatriptan, one received oral rizatriptan and one received oral eletriptan. One patient had no pain (visual analog scale [VAS] ≤ 4), 17 patients had moderate pain (VAS > 4 and < 8) and 6 patients had severe pain (VAS ≥ 8). A positive response, based on at least 50% reduction of the headache within 2 h based on the VAS was reached in 21 patients (87.5%), and 11 patients (45.8%) were completely pain-free after 2 h. There was neither a difference in reduction of headache intensity after treatment between migraine with-(MA) or without (MO) aura (*P* = 0.42), nor an effect of age (*P* = 0.51). None of the features related to the patient’s health were significantly associated with response to treatment (Table 1).

**Table 1:** Descriptive statistics study cohort and their association with treatment effect.

	Mean (SD)	N (yes/no)	*P* value*
Age	37.6 (10.9)		0.95
BMI	23.2 (3.0)		0.87
Migraine attacks/month	4.3 (4.2)		0.43
Heart rate	68.4 (8.0)		0.88
Blood pressure			
Systolic	123 (11)		0.86
Diastolic	77 (9)		0.44
Medication use			
Prophylactic migraine		3/24	0.62
Acute migraine		22/2	0.94
Concomitant		12/12	0.52
Medical history			
Psychiatric		8/16	0.45
CNS		5/18	0.93
Cardiopulmonary		4/20	0.84
Endocrine		1/23	*NA*
Gastrointestinal		3/21	0.79
Urogenital		5/19	0.91
Gynecological		6/18	0.67
Musculoskeletal		8/15	0.70
Provoking factors			
Physical activity		10/14	0.42
Light		13/11	0.58
Stress		21/3	0.61
Menstruation		15/8	0.37
Alcohol		12/11	0.69
Fragrances		9/15	0.40
Sleep deprivation		19/4	0.11
Sleep excess		19/4	0.11

*Association with reduction in headache score (VAS) using a t-test (binomial features) or linear regression (continuous features).

**Figure 2:**
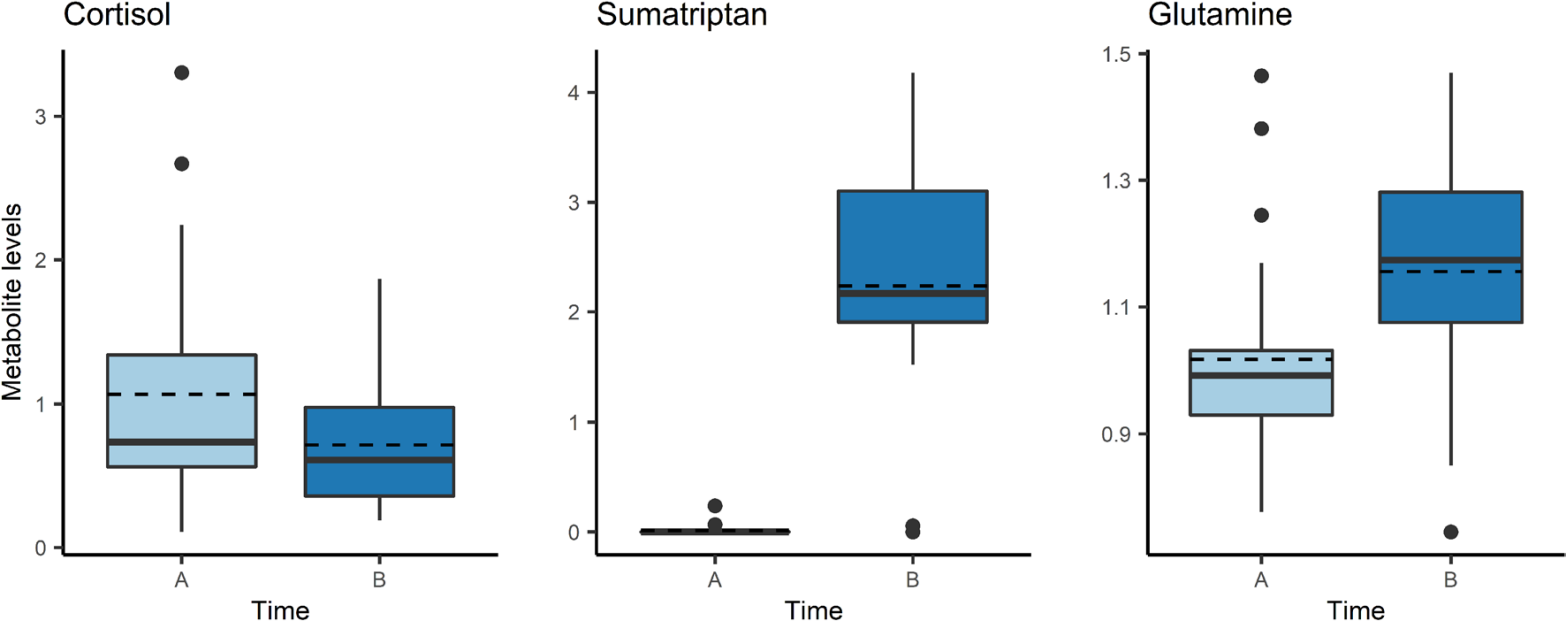
Differential metabolites during migraine attack (Timepoint A) versus 2 h after treatment with a triptan (Timepoint B). Mean values are indicated by dashed line.

Comparing samples from the migraine attack before treatment (A) with 2 h after treatment (B), we detected three differential metabolites (*P*_adj_ < 0.05): cortisol, sumatriptan and glutamine (Fig. 2). None of these were differential during the cold pressure test and are therefore, likely treatment- or migraine-specific. Results from the differential metabolomic analysis are presented in Supplementary Table 1. Using correlation and regression analyses, we further analyzed the three differential metabolites.

### Cortisol

The level of cortisol was lower 2 h after treatment (0.71 [SD = 0.46]) than before treatment (1.07 [SD = 0.77]) (*P*_adj_ = 0.04). The cortisol levels estimated by LC–MS/MS were confirmed by steroid profiling (r_s_ = 0.95, *P* = 2.20 × 10^–16^). It was not significantly higher in headache-free patients than in patients still suffering from headache, 2 h after treatment (i.e., 0.90 [SD = 0.48] vs. 0.56 [SD = 0.39], *P* = 0.06). Cortisol levels correlated significantly with several glycerophosphocholines (LysoPC16:0 [r_s_ = -0,55], LysoPC20:4 [r_s_ = − 0.55], and LysoPC22:6 [r_s_ = 0.51]; *P*_adj_ < 0.05). Using the RNA-sequencing data, we found that cortisol levels were significantly correlated with *DDIT4* (DNA Damage Inducible Transcript 4; r_s_ = 0.78, *P*_adj_ < 0.05). *DDIT4* was also differentially expressed after treatment (*P* = 1.73 × 10^–6^) and correlated with reduced blood cortisol levels after treatment with triptan, as published previously^7^.

### Sumatriptan

**Figure 3:**
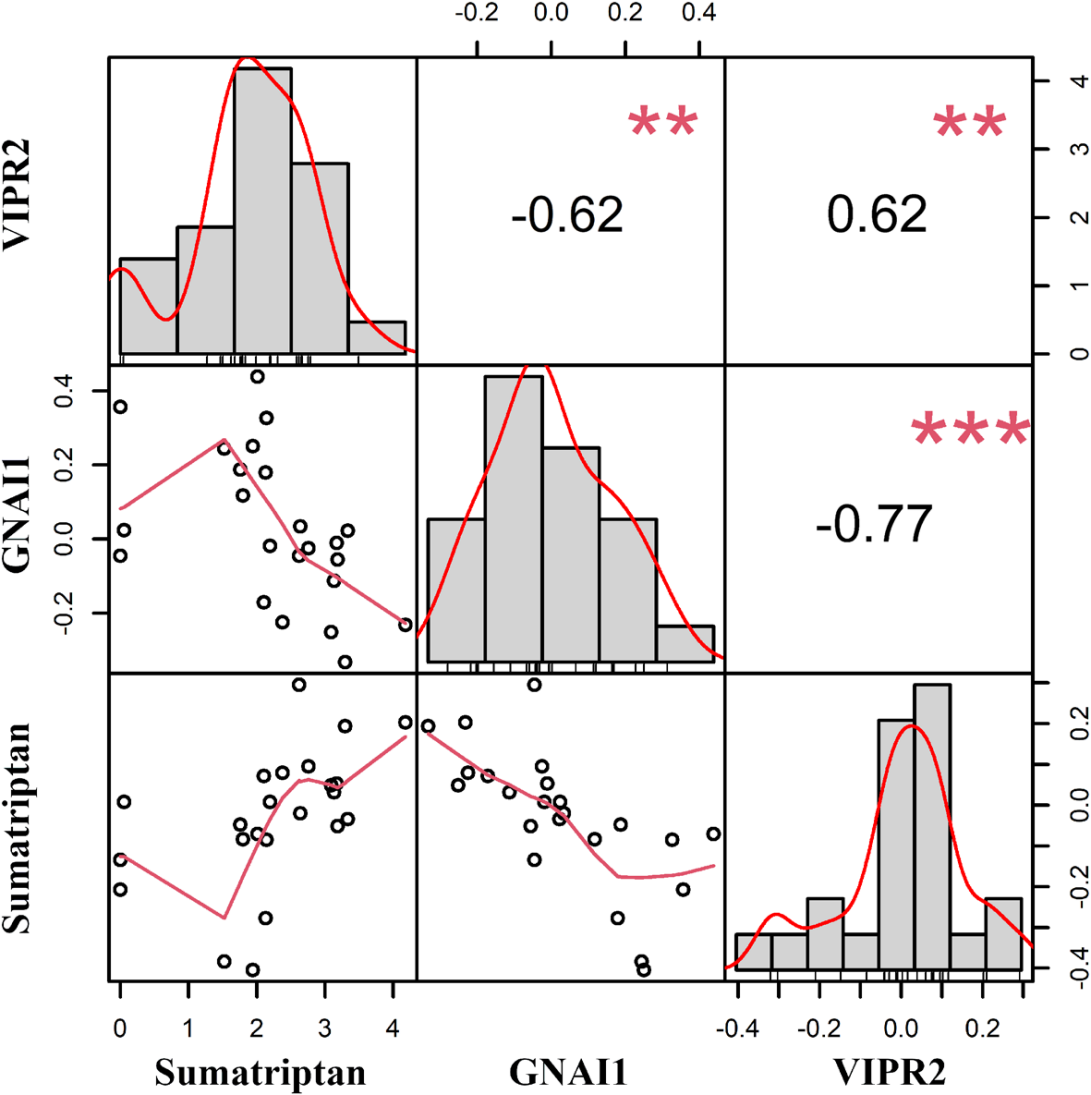
Visualization of correlation matrix of sumatriptan with genes known to be involved in cAMP regulation, i.e., *GNAI1* and *VIPR2*. Above the diagonal the absolute value of the correlation with significance (**p < 0.01, ***p < 0.001), on the diagonal histograms of the change in expression of VIPR2, GNAI1 and sumatriptan, and below the diagonal the bivariate scatterplots. Note that values on both y- and x-axis are change in levels/expression after treatment with sumatriptan.

Sumatriptan, as expected, was practically absent before treatment (0.01 [SD = 0.05]) but was highly increased after treatment (2.24 [SD = 1.07]) (*P*_adj_ = 0.02); excluding the two patients receiving another triptan (having 0 and 0.06 expression of sumatriptan) resulted in a mean expression of 2.42 (SD = 0.87). To gain insight into the potential metabolism or mechanism of action of sumatriptan, we investigated whether the level of sumatriptan correlated with the change of any metabolites or genes. The level of sumatriptan was dominated by zero’s (i.e., before treatment), therefore, we used the change in sumatriptan levels (after treatment–before treatment) to find genes/metabolites being affected by the intervention with sumatriptan. Change of palmitoylcarnitine (r_s_ = 0.65) and diphenylalanine (r_s_ = 0.66) levels were positively correlated with increased levels of sumatriptan (*P*_adj_ < 0.05). The change in level of sumatriptan showed a negative correlation with change in expression of *TRAPPC11* (Trafficking protein particle complex subunit 11; ρ = − 0.74), *ABTB2* (Ankyrin repeat and BTB domain containing 2, r_s_ = − 0.66), *RHBDD1* (Rhomboid Domain Containing 1; r_s_ = − 0.65), *ZNF443* (Zinc finger protein 443, r_s_ = 0.62), *GNAI1* (G protein subunit alpha I1, r_s_ = − 0.62), *ANOS1* (Anosmin 1, r_s_ = − 0.61) and *NRN1* (Neuritin 1, r_s_ = − 0.61), and a positive correlation with change in expression of *FZD4* (Frizzled class receptor 4, r_s_ = 0.68), *ZNF114* (Zinc finger protein 114, r_s_ = 0.67), *PIWIL3* (Piwi like RNA-mediated gene silencing 3, r_s_ = 0.66), *NRIP3* (Nuclear receptor interacting protein 3, r_s_ = 0.64), *TMEM210* (Transmembrane protein 210, r_s_ = 0.64), *KRT8* (Keratin 8, r_s_ = 0.63), *VIPR2* (Vasoactive intestinal peptide receptor 2, r_s_ = 0.62), and *KLHL30* (Kelch like family member 30, r_s_ = 0.62); all *P*_adj_ < 0.05. None of the genes were previously detected in differential expression analysis^7^. Genes known to be important for regulation of cAMP levels are highlighted in Fig. 3. All correlations are visualized in Supplementary Fig. 2. Interestingly, *ZNF443* is correlated with both *VIPRR2* and *GNAI1*.

### Glutamine

Glutamine levels were higher after treatment with a triptan (1.16 [SD = 0.17) than before treatment (1.02 [SD = 0.16]) (*P*_adj_ = 0.04). Glutamine correlated significantly (*P*_adj_ < 0.05) with gamma-glutamyl glutamine (γ-Glu Gln, r_s_ = 0.67), O-acetylcarnitine (r_s_ = 0.59) and acetylcarnitine (r_s_ = 0.63), hydroxybutyrylcarnitine (r_s_ = 0.63), gamma-glytamyl-2-aminobutyric acid (r = 0.55). O-acetylcarnitine and acetylcarnitine were very similar in structure and highly correlated to each other (r_s_ = 0.85, *P* = 2.20 × 10^–16^). No genes were significantly correlated with glutamine; however, we fitted the gene expression of top features detected by Qlattice on glutamine expression. Genes associated with glutamine were *CHD7* (Chromodomain Helicase DNA binding protein 7)*, SCN3A* (Sodium Voltage-gated channel alpha subunit 3), *DLC1* (DLC1 Rho GTPase-acitvating protein), *HIVEP1* (HIVEP Zinc Finger 1), and *PDE1C* (Phosphodiesterase 1C). Several of the predicting genes were previously detected as differentially expressed after treatment: *CHD7* (*P* = 2.73 × 10^–4^), *SCN3A* (*P* = 6.14 × 10^–4^), *DLC1* (*P* = 4.23 × 10^–4^), *HIVEP1* (*P* = 5.85 × 10^–3^) and *PDE1C* (*P* = 0.03).

## Discussion

In this study, three differential metabolites were detected using untargeted metabolomics comparing a spontaneous migraine attack before and after treatment with triptan: cortisol, sumatriptan and glutamine. Sumatriptan was only present after treatment and not during migraine attack. Besides finding cortisol to be reduced and glutamine to be increased 2 h after treatment, multi-omics assessment gave novel insight into molecular mechanisms of a spontaneous migraine attack treated with triptan.

The study design is novel, presenting sequential sampling and a multi-omics approach. Migraine attacks come irregularly and occur outside the hospital; therefore, collection of large sample sizes is challenging. Recently, *Aczél *et al.^20^ compared samples in- and outside a migraine attack, and subsequently, compared them to healthy controls. Based on findings without correction for multiple tests, they found changes related to mitochondrial dysfunction and altered inflammation and cytokine pathways among migraine patients, compared to healthy controls. We previously did not find any changes in gene- or metabolite expression in similar comparisons using a comparable set of samples after multiple-testing correction, and were not able to replicate findings of *Aczél *et al. In those comparisons, the time interval in combination with small sample size is not giving enough power to detect changes. In this study, two samples were taken during migraine attack, one before and one two hours after treatment with triptan. The paired-sample design increases the statistical power as variability is reduced by removing influence of any stable individual differences (e.g., medication use or migraine characteristics). With this short time interval between samples we previously found significant changes in gene expression profiles^7^. We now similarly show distinct metabolomic changes and integrate those with the transcriptomic data to unravel molecular mechanisms involved in migraine and/or its treatment. As the (spontaneous) migraine attacks of all patients was treated with a triptan, and neither a group of patients without treatment nor a group of patients treated with triptan without having a migraine attack were included in this clinical trial, we were not able to distinct whether ‘omic changes were migraine-related and/or triptan related.

### Reduced levels of cortisol after treatment

Cortisol levels are similar in migraine patients and controls^21^. Here, we found reduced cortisol 2 h after treatment compared to before treatment. It could be expected that a stress hormone, like cortisol, is raised during a migraine attack followed by a lower level when the headache disappears. Even though we did not find the metabolite to be up-regulated after the cold-pressor test, i.e., a general pain/stress test, we cannot eliminate this explanation as the cold pressor test might have had a short(er)-lasting effect on cortisol or no difference was found due to lack of statistical power.

### Sumatriptan detected in blood after treatment

Even though the study design did not provide the opportunity to distinguish whether ‘omic changes were due to migraine and/or triptan treatment, the detection of sumatriptan using untargeted metabolomics gave the opportunity to investigate sumatriptans mechanism of action by integrative analysis with the full transcriptomic profile. Integration of the change in sumatriptan with transcriptomics showed a correlation with *GNAI1*, encoding the G Protein subunit alpha I1, inhibiting adenylate cyclase activity via *ADCY5,* and subsequently leading to decreased levels of cAMP. In addition, we found a correlation with *VIPR2*, encoding a vasoactive intestinal peptide (VIP) receptor, which similar to *GNAI1* regulates cAMP via *ADCY5*. Migraine attacks can be provoked by substances, of which several cause upregulation of cAMP, i.e., CGRP^22^, PACAP^23^ and cilastozol^24^. Moreover, CGRP monoclonal antibodies are a novel treatment for migraine. Several mechanisms are proposed for this successful treatment, one of them is the downregulation of cAMP^25^. It is known that sumatriptan is binding to the 5-HT_1B/1D_ receptors that are coupled to G protein subunit alpha receptors (e.g., GNAI), so we here show a direct effect of sumatriptan (measured by LC–MS/MS) on gene expression (measured by RNA-Sequencing). The correlation of *ZNF443*, known to regulate gene transcription, with both *GNAI1* and *VIPR2* implies a role for *ZNF443* in cAMP regulation.

Among the metabolites, we found a positive correlation between sumatriptan and palmitoylcarnitine. Palmitoylcarnitine plays a key role in the transport of long-chain fatty acids into the mitochondria for energy production. This is in line with our previous investigation of changes in gene expression during the triptan-treated migraine attack: several differentially expressed genes functioned in fatty acid oxidation. We previously linked fatty acid oxidation, based on existing literature, to migraine pathophysiology, but here, we suggest a direct association with treatment. To our knowledge, it is not known how sumatriptan might affect fatty acid oxidation.

### Increased levels of glutamine after treatment

Lastly, we saw increased levels of glutamine 2 h after treatment with a triptan. Glutamine is an α-amino acid used in the biosynthesis of proteins. It is metabolized into glutamate, the most abundant excitatory neurotransmitter in the central nervous system. Several studies have investigated the importance of the glutamatergic system in migraine^26^^[,27^, and migraine patients overusing triptans had reduced levels of glutamate^28^. Sumatriptan inhibits the glutamatergic synaptic transmission and thereby reduced neurotransmitter release^29^. The increase in glutamine might be a feedback response where levels of glutamine are increased to balance the glutamatergic system. This hypothesis is strengthened by the finding that the increase of glutamine was positively correlated with γ-Glu Gln, a dipeptide of glutamine joined to the gamma-carbon of glutamate. Glutamine has also an important role in the immune system, being a fuel source for immune cells, so an increase of glutamine during the migraine attack might indicate an increased activity of the immune system*.* Among the correlated metabolites, we found several carnitine’s (i.e. O-acetylcarnitine, acetylcarnitine and hydroxybutyrylcarnitine) supporting again the importance of the fatty acid oxidation in migraine and/or treatment of migraine.

## Conclusions

As the study, due to both ethical and practical limitations, was not a crossover design, we could not distinguish whether changes in ‘omics were due to migraine or due to treatment. However, the detection of sumatriptan using untargeted metabolomics gave the opportunity to investigate its working mechanisms. Here, we showed its effect on regulation of cAMP levels, a key mechanism of triptans. The upregulation of glutamine after treatment, may be the result of the migraine attack or due to treatment with triptan. This study shows the importance of cAMP and fatty acid oxidation in the molecular mechanisms of sumatriptan.

## Supplementary Information

Supplementary Table S1.

Supplementary Table S2.

Supplementary Figure S1.

Supplementary Figure S2.

## Data Availability

Annotation of differential and predictive metabolites is presented in Supplementary Table 2. The metabolomic dataset supporting the conclusions of this article are available at: The mass spectral molecular network data: https://gnps.ucsd.edu/ProteoSAFe/status.jsp?task=c5abc8dea50e4b27a383a2fb2d0bef18. MS2LDA substructure information: https://gnps.ucsd.edu/ProteoSAFe/status.jsp?task=4b0effdcf8654a9c96eb9bbe07d61ea7. Network Annotation Propagation in silico structure annotations: https://proteomics2.ucsd.edu/ProteoSAFe/status.jsp?task=d3af9ae0f4154f9dacf06d4768cfa704 and https://proteomics2.ucsd.edu/ProteoSAFe/status.jsp?task=79ea689901f6471eb9f77688729db577. The transcriptomic dataset supporting the conclusions of this article is available in the European Genome-Phenome Archive (EGA), under EGA ID: EGAS00001006795 [https://ega-archive.org/studies/EGAS00001006795].

## Abbreviations

cAMP: Cyclic adenosine monophosphate
ECG: Electrocardiography
ERK: Extracellular signal-regulated kinases
GNPS: Global Natural Products Social Molecular Networking Platform
LC–MS/MS: Liquid chromatography-tandem mass spectrometry
MA: Migraine with aura
MO: Migraine without aura
P_adj_: Adjusted p-value
r_s_: Rho
VAS: Visual analog scale
