# Supplementary material for: Multi-omic analyses of triptan-treated migraine attacks gives insight into molecular mechanisms
Source: Sci Rep. 2023 Jul 31;13:12395. doi: 10.1038/s41598-023-38904-1 (PMC10390468; doi:10.1038/s41598-023-38904-1)

Supplementary figrue 1. Spectral mirror plots. Spectral mirror plots of metabolite annotations retrieved from GNPS with a spectral similarity score (cosine score) >=0.8.
Spectra were extracted automatically using the Metabolomics Spectrum SResolver (http://metabolomics-usi.ucsd.edu) and mirror plots were produced in Python 3.7 using the spectrum_utils package [30].
Experimental spectra are displayed in the top panel whereas library spectra are displayed in the bottom panel. Matchin peaks are colored black and green for the experimenatl and library spectra, respectively, and non-matchin peaks are colored grey.

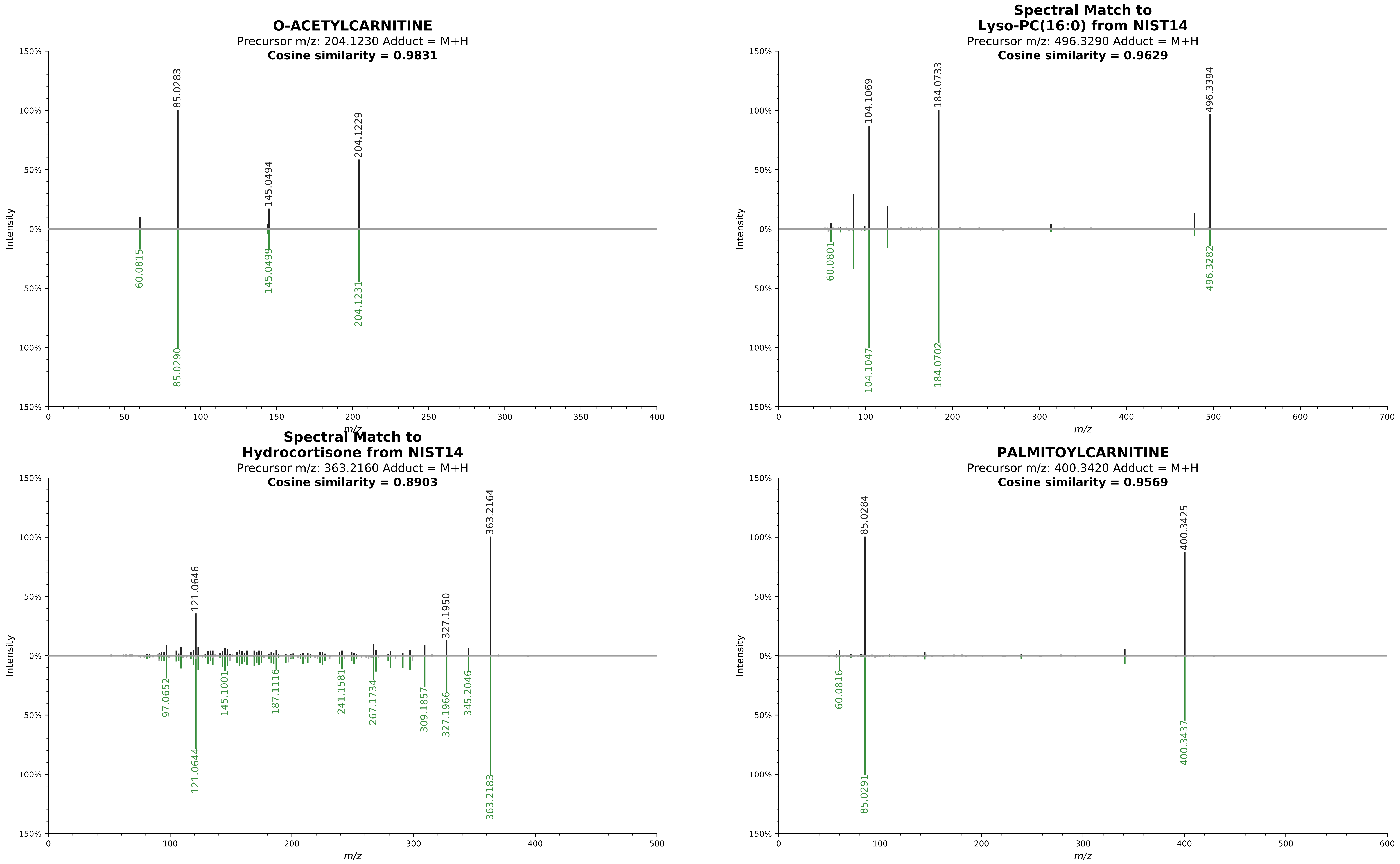

Supplement: Supplementary file 3 — Supplementary Figure S1. [file 41598_2023_38904_MOESM3_ESM.pdf]
